# T Helper Lymphocyte Subsets and Plasticity in Autoimmunity and Cancer: An Overview

**DOI:** 10.1155/2015/327470

**Published:** 2015-10-25

**Authors:** Ekaterina A. Ivanova, Alexander N. Orekhov

**Affiliations:** ^1^Department of Pediatric Nephrology and Growth and Regeneration, Katholieke Universiteit Leuven and University Hospitals Leuven, 3000 Leuven, Belgium; ^2^Laboratory of Angiopathology, Institute for General Pathology and Pathophysiology, Moscow 125315, Russia; ^3^Institute for Atherosclerosis Research, Skolkovo Innovative Center, Moscow 121609, Russia; ^4^Department of Biophysics, Faculty of Biology, Lomonosov Moscow State University, Moscow 119991, Russia

## Abstract

In response to cytokine signalling and other factors, CD4-positive T lymphocytes differentiate into distinct populations that are characterized by the production of certain cytokines and are controlled by different master transcription factors. The spectrum of such populations, which was initially limited to Th1 and Th2 subsets, is currently broadened to include Th17 and Treg subsets, as well as a number of less studied subtypes, such as Tfh, Th9, and Th22. Although these subsets appear to be relatively stable, certain plasticity exists that allows for transition between the subsets and formation of hybrid transition forms. This provides the immune system flexibility needed for adequate response to pathogens but, at the same time, can play a role in the pathogenic processes in cases of deregulation. In this review, we will discuss the properties of T lymphocyte subsets and their plasticity, as well as its implications for cancer and autoimmune diseases.

## 1. Introduction

T helper (Th) lymphocytes play a key role in the adaptive immune system exerting a wide spectrum of biological functions. CD4+ T cells regulate both cytotoxic cellular immune response and B cell-dependent antibody production; they interact with the components of the innate immune system and respond to stimuli from the antigen-presenting dendritic cells. Naïve CD4+ cells can be activated by the encounter with antigen via peptide/MHC class II TCR and differentiate into T effectors and long lasting memory T cells. Depending on the intensity of stimulation and presence of certain cytokines and other factors, CD4+ T cells can differentiate into various subpopulations of T cells with specific functions and properties [1]. This functional specialization is regulated by a number of transcription factors that are activated in response to specific stimuli and promote the expression of distinct patterns of soluble factors and surface molecules. These patterns can be used for identification of different classes of T lymphocytes.

CD4+ T helper cells deriving from thymus differentiate at the periphery in response to antigen stimulation [2]. The first classification divided CD4+ effector cells into two subsets, Th1 and Th2 [3]. Th1 cells are induced in response to pathogens, such as viral infections, and are characterized by the production and release of interferon gamma (IFN-*γ*). They promote the activation of macrophages that are efficient against intracellular pathogens. Th2 cells are mostly involved in humoral immune response and provide help to B cells to produce class-switched antibodies.

**Figure 1:**
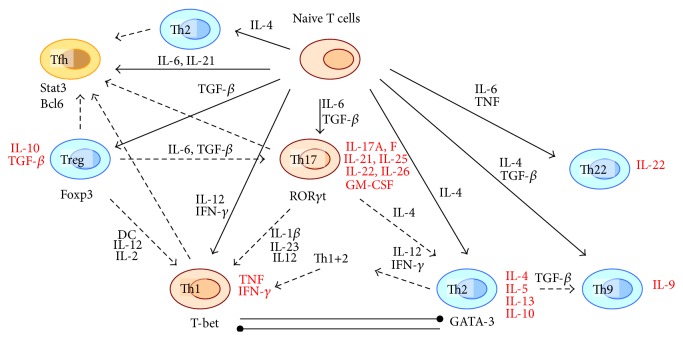
Simplified scheme of T cell differentiation pathways and plasticity (dashed arrows). Secreted cytokines are listed in red. IL: interleukin; DC: dendritic cells; GM-CSF: granulocyte macrophage colony-stimulating factor; TGF-*β*: transforming growth factor beta; TNF: tumor necrosis factor; IFN-*γ*: interferon gamma.

In the recent years, it became evident that more functional subsets of T helper cells can be induced by various stimuli* in vivo* and* in vitro*. IL-17-producing CD4+ T cells differentiated in response to transforming growth factor beta (TGF-*β*) and certain interleukins were recognized as a distinct subset of Th17 cells [4]. Another subset of CD4+ T lymphocytes are the regulatory T cells (Tregs) expressing the transcription factor Foxp3 [5]. Follicular T helper (Tfh) cells have been proposed as a distinct lineage of T helper cells that resides in follicles and assists B cells to generate antibodies [6, 7]. Other subsets of T lymphocytes have been identified based on the production of different cytokines, such as Th9 and Th22 (expressing IL-9 and IL-22, resp.) [8–10]. Detailed study of these subsets became a subject of current research, and the recent findings on Th9 and Th22 cells have been summarized in excellent reviews [11, 12]. A simplified scheme of T lymphocyte subsets is presented in Figure 1.

The differentiation of various subsets of T cells depends on switching the specific genetic programs responsible for the expression of cytokine and receptor patterns. Presence of certain transcription factors is considered as a marker of lymphocyte subsets, and these factors can regulate themselves and one another creating positive or negative feedback loops and setting the differentiation conditions [13]. Another mechanism of differentiation is the epigenetic control [14, 15]. Epigenetic regulation, which does not affect the DNA sequence but consists of various chromatin modifications, such as nucleosome positioning, histone modification, and DNA methylation, was shown to play an essential role in T cell generation, differentiation, and plasticity [16].

Detailed studies of T lymphocyte behavior* in vivo* and* in vitro* demonstrated that T lymphocyte subsets are characterized by certain flexibility and can change their functional phenotypes and cytokine and receptor expression patterns in response to milieu changes. Moreover, such plasticity plays an important role in the initiation and development of pathological processes, including cancer and autoimmune diseases. In this review, we will briefly characterize the main subsets of T lymphocytes that have been described so far, their plasticity, and its association with human pathologies.

## 2. T Lymphocyte Subsets

### 2.1. Th1 Cells

Th1 cells are induced in response to IFN-*γ* and IL-12, which plays a key role linking the innate immunity and adaptive immunity and is secreted primarily by the dendritic cells. IFN-*γ* and IL-12 signals are mediated by Stat1 (signal transducer and activator of transcription 1) and Stat4. Th1 cells express the master transcription factor T-bet encoded by the* Tbx21* gene and are characterized by the production of IFN-*γ*, which also reinforces the Th1 polarization, creating a positive feedback loop, and suppresses the alternative differentiation programs [17, 18]. The early IFN-*γ* that drives the differentiation of naïve T cells towards the Th1 phenotype can be produced by activated natural killer (NK) cells [19]. The relative stability of Th1 phenotype can be partly explained by a self-supporting transcriptional circuitry, because T-bet can induce its own expression either directly or indirectly and suppress the alternative transcription factor GATA-3, responsible for Th2 differentiation [20–22].

### 2.2. Th2 Cells

Th2 cells are induced in the presence of IL-4, which antagonizes Th1 polarization, via Stat6 signalling. Their master regulator transcription factor is GATA-3, which is also capable of self-activation, providing a self-reinforcing feedback [23]. GATA-3 and T-bet are characterized by mutual antagonism, which favors the polarization of T cells towards either Th1 or Th2 states depending on the surrounding cytokine profile and makes the transition states unstable [13]. Th2 cells express the signature cytokines IL-4, IL-5, and IL-13 and are involved into humoral immune responses to extracellular infectious agents and parasites [24, 25]. They are also implicated in the development of allergic reactions and atopy [26].

### 2.3. Th17 Cells

Th17 cells are currently recognized as an independent T cell lineage in addition to Th1 and Th2 [27–29]. Th17 polarization occurs in the presence of IL-6 or IL-21 and TGF-*β* [30, 31]. Their differentiation is independent from the transcription factors T-bet and GATA-3 and the related signalling but regulated by Stat3 and Smad pathways and retinoic acid receptor-related orphan receptors ROR*γ*t (RORc in humans) and ROR*α* [32–34]. These cells are producing IL-17A and the related IL-17F. They also express other cytokines including IL-21, IL-22, and granulocyte macrophage colony-stimulating factor (GM-CSF) [1]. IL-23 is important for Th17 survival and inflammatory potential and plays a role in human autoimmune pathologies [35]. It has been demonstrated that TGF-*β* and IL-21 drive the differentiation of Th17 cells from naïve CD4+ cells and IL-23 and IL-1*β* induce their differentiation from memory T cells [31, 36]. Th17 cells are present in normal circumstances, especially in the gut, where they provide protection against bacterial and fungal infections, but are upregulated both in the gut and in other tissues during inflammation [37–39].

### 2.4. Treg Cells

Tregs are a subset of T cells controlled by the master transcription factor Foxp3 and differentiated in response to TGF-*β* [40]. However, Foxp3 is also expressed by different nonregulatory activated T cells in humans, and analyzing other markers is needed for identification of Treg cells. It has been demonstrated that the expression of CD127 (IL-7R) is suppressed in Treg cells, and hence the low level of CD127 could be used as a relevant marker of this subset, allowing for distinguishing them from activated effector T cells. A higher expression level of folate receptor 4 has also been proposed as a marker of Treg cells [41]. The stability of Treg subset is dependent on its origin. Tregs derived from the thymus are considered to be a stable subset. On the other hand, Tregs can also be induced at the periphery in response to TGF-*β* and antigen presence resulting in the formation of adaptive or inducible Tregs (iTregs) [42]. These cells were shown to be less stable in their functional phenotype. In naïve CD4+ T cells, TGF-*β* induced both Foxp3 and ROR*γ*t, but the former is dominant and suppresses ROR*γ*t in the absence of IL-6, shifting the balance from Th17 to iTreg in inflammatory conditions [13, 43]. There is functional similarity between the natural and inducible Tregs, but they appear to be different in their epigenetic status [44]. A surface marker of Treg cells is the IL-2 receptor alpha chain (CD25), and IL-2 is important for their survival and homeostasis [45]. Treg cells play the key role in maintaining the peripheral tolerance. They can suppress the function of other effector T cells and antigen-presenting cells by cell-cell interactions and the release of suppressive cytokines, such as TGF-*β* and IL-10 [46–48]. The population of Treg cells is heterogeneous by the expression of various surface markers and can be subdivided into several subtypes, notably, memory-like (generated upon antigen encounter) and naïve-like Tregs [47]. Treg dysfunction was shown to be associated with various autoimmune pathologies, including multiple sclerosis, type I diabetes, psoriasis, and myasthenia gravis [49–51].

### 2.5. Th9 Cells

A population of IL-9 producing cells has first been described in the late 1980s [52]. Later it was demonstrated that stimulation of Th2 cells with TGF-*β* or naïve T cells with IL-4 and TGF-*β* can lead to generation of cells, positive for IL-9 but not for IL-4, indicative of the existence of a distinct subset of T helper cells, termed Th9 [8, 9]. The differentiation and function of Th9 cells, as well as their possible role in autoimmune diseases and allergy, have recently been described in an excellent review [53]. Generation of Th9 cells from naïve CD4+ T cells is stimulated by the addition of TGF-*β* and further enhanced by IL-4, although an IL-4-independent IL-9 production is possible in the presence of IL-2, another cytokine essential for Th9 differentiation. Other cytokines, including IL-1*α*, IL-1*β*, IL-33, IL-21, and IL-25, also promote IL-9 production, whereas IL-27 suppresses it [11]. The accumulating evidence indicates that Th9 subset exists* in vivo*. Elevated IL-9 production and Th9 differentiation have been demonstrated in mouse models of allergy and melanoma [54, 55]. However, IL-9 can be produced by multiple cells* in vivo*, and innate lymphoid cells (ILCs) are the main detectable source of this cytokine in studied models [56]. IL-9 has a number of important functions in the immune system: it promotes the survival and proliferation of T cells and mast cells, stimulates the production of several cytokines, and modulates B cell responses. It has also effects on some nonhematopoietic cell types. Elevated production of IL-9 plays an important role in autoimmune processes, allergy, and antitumor immunity [11]. Th9 cells also produce IL-10 and IL-21, although their functions remain to be elucidated.

### 2.6. Th22 Cells

IL-22 is a member of IL-10 family and has multiple functions, targeting epithelial and pancreatic cells, hepatocytes, and some types of fibroblasts, mediating host defence against invasive pathogens [12]. Like IL-9, IL-22 can be produced by various types of activated T cells, including Th17, CD8+ cells, and innate immune cells. T cells expressing IL-22, but not IL-17 or IFN-*γ*, have been described in humans leading to identification of Th22 as a distinct subset of T cells [57, 58]. Differentiation of Th22 from naïve CD4+ T cells is induced by TNF-*α* and IL-6 and further promoted by IL-1*β*. Another way of Th22 generation, not completely dependent on TNF and IL-6, has also been reported [59]. The production of IL-22 is increased in several autoimmune diseases, such as inflammatory bowel disease, allergic asthma, systemic sclerosis, and rheumatoid arthritis, where it can play both protective and pathogenic roles depending on the context and the disease phase [12]. Th22 cells can influence mesenchymal and epithelial cells and play a role in the development of skin inflammation, such as psoriasis and atopic dermatitis [60]. Increased Th22 cells and IL-22 were shown to be associated with various tumors, and several lines of evidence indicate an important role of this T cell subset in tumorogenesis [61–63]. Therefore, Th22/IL-22 can be regarded as a potential target of antitumor therapy [12].

### 2.7. Tfh Cells

A subset of T helper cells residing in B cell follicles (Tfh) has been described [64, 65]. These cells play an important role in maintaining of B cell memory and the antibody production. They express IL-21 similar to Th17 cells, and their differentiation is regulated by Stat3 and also Bcl-6 [66, 67]. However, unlike other CD4+ T lymphocytes that constantly migrate through folliculi, Tfh cells dwell there, likely because of the expression of CXCR5 chemokine receptor, which is currently considered as the best surface marker available for this subset. The relationship of Tfh and other T helper cell subsets is currently unclear, as these cells may represent not a distinct subset, but rather a functional state of other subsets with follicular location.

## 3. Mechanisms of T Cell Plasticity

Recent studies indicate the existence of certain flexibility of T cell commitment. T cell subsets are traditionally defined by the cytokine pattern that they produce, the transcription factors that regulate their functions and, in some cases, the expression of specific chemokine receptors. Tissue microenvironment also plays an important role in differentiation and function of Th cell subsets [68].

T cell subsets defined by the expression of CD4 or CD8 or different types of T cell receptors are determined during their development in the thymus and are inflexible. At the same time, subsets that are defined by the activation of transcription factors (and hence the different genetic programs) in the periphery can demonstrate plasticity [69]. There are, however, some rules that regulate the transition between the T cell subsets. Th1 and Th2 subsets appear to be most stable, as both of them are regulated by mutually suppressing and self-reinforcing transcription and signalling factors (T-bet and IFN-*γ* for Th1 and GATA-3 and IL-4 for Th2) [13]. Moreover, cells that express the IL-12 receptor remain responsive to IL-12 signalling, and Th17 cells can undergo an IL-12-dependent transition to Th1 state in mice and humans [70–72]. The IL-12 receptor plays therefore a central role in the described transitions and the proinflammatory response. The IL-12 receptor consists of two chains, IL-12R*β*1 and IL-12R*β*2 [73]. The intensity of IL-12 signalling is limited by the availability of the IL-12R*β*2 chain, as its expression is significantly less than that of IL-12R*β*1 [74]. It has been demonstrated that even stably committed Th2 cells can reexpress the IL-12R*β*2 and produce IFN-*γ* together with IL-4* in vivo* in response to viral infections [75]. A Th1+2 hybrid transition state has also been observed, which is induced by type I interferons in combination with IFN-*γ* and IL-12 [13, 76].

Th2 cells were demonstrated to convert to Th9 cells in response to TGF-*β* [8]. It has been also demonstrated that a considerable portion of Th9 cells can acquire Th1 phenotype and produce IFN-*γ in vivo* [77, 78]. Th17 subset, apart from the conversion to Th1 phenotype, can also acquire Th2-type IL-4-expressing phenotype, as demonstrated in a helminthic infection model [79]. iTreg subset, which appears to be less stable than thymus-derived Treg, is susceptible to transition towards Th17 in the presence of IL-6 in inflammatory environment [13]. The possibility of Treg transition to Th1-like phenotype coexpressing Foxp3 and T-bet has been demonstrated in mice [80]. T-bet induction in Treg cells has been observed in infection models and colitis [81, 82]. Detailed studies demonstrated the presence of natural Tregs capable of inducing T-bet and IFN-*γ* expression resulting in a Th1-like phenotype [83]. Such Treg-Th1 plasticity is dependent* in vitro* on IL-12 and IL-2 and might play a role in autoimmune diseases. Studies in mice and humans have demonstrated that Tregs are also capable of becoming Th17 cells in the presence of IL-6 and TGF-*β* [84, 85]. Recently, differentiation properties of CXCR3-relative chemokines have been described: CXCL10 was shown to polarize effector Th1 cells and CXCL11 to promote differentiation of Tregs from naïve T cells and CXCR3+CD4+ effector T cells, associated with experimental allergic encephalomyelitis (EAE) [86].

On the other hand, some transitions between subsets apparently do not occur, including Th2 to Th17 or Treg transition or Th1 to Treg or naïve T cells [13]. The plasticity of T cell fates can be advantageous for host defence against pathogens but can also play a role in pathological processes, including autoimmune diseases and cancer.

## 4. Plasticity in Cancer

Regulation of the immune response in cancer receives much attention as possible instrument for the development of novel antitumor therapies. The developing tumor induces an immune reaction driven by the cells of the innate immune system (innate lymphoid cells (ILCs), NKT, *γδ* T, NK, and macrophages). Cytotoxic T lymphocytes and IFN-*γ*-producing CD4+ T cells are recruited to the tumor and induce cell death, further activation of NK and macrophages, and inflammation. Th1 and Th17 subsets play an important role in the antitumor response, producing the inflammatory cytokines and assisting the cell-mediated killing of tumor cells [87]. These responses, however, can be suppressed by Treg cells that are also recruited by the growing tumor [88]. Tregs are powerful inhibitors of antitumor immunity and represent the greatest obstacle to immunotherapy of cancer [89]. At the early stages of the process, Tregs are concentrated in the tumor mass, locally inhibiting the effector immune responses and allowing the tumor to progress. The ratio of Treg to T effector cells in the tumor mass has therefore a prognostic value [90]. At later stages, Tregs can be upregulated systemically, suppressing the immune protection against metastases [91]. It has been demonstrated that systemic Treg depletion induced regression of melanoma metastases [92]. Combination of Treg depletion with immunogene stimulation was highly effective against weakly immunogenic sarcomas in mice [93]. Therefore, regulation of Tregs in cancer, especially locally in tumors, appears to be a promising therapeutic option, and several Treg-suppressing agents have been already developed [94, 95]. T cell plasticity mechanisms can also be exploited for this purpose. Different approaches could be taken to shift the balance towards Th17 rather than Treg differentiation, such as agonists for retinoic acid receptors or direct introduction of ROR*γ*t [96]. Blocking TGF-*β* by specific antibodies prevented the peripheral induction of Tregs and reduced the tumor burden in mice. This approach, however, is associated with the risk of autoimmune disorders [97]. Cyclophosphamide antitumor effect is partly dependent on modulation of the immune response promoting Th1 and Th17 cells. A recent study on mouse cancer models demonstrated that gut microbiota was indispensable for cyclophosphamide-induced generation of Th17 cells with antitumor activity. Treatment with the drug induced some gram-positive bacteria translocation into secondary lymphoid organs where they stimulated differentiation of “pathogenic” IFN-*γ*-producing Th17 lymphocytes [98].

The role of Th17 cells in cancer remained controversial for a long time [99]. It has been demonstrated that Th17 cells infiltrate tumors and their concentrations there are highly elevated in comparison with surrounding tissues implying a specific role in tumor development [100–102]. Such accumulation of Th17 cells was associated with improved patient survival in some cancer types and with poor prognosis in other types [103, 104]. Interestingly, some observations indicate that Treg cells infiltrating tumors can be converted to the proinflammatory Th17 phenotype in some cancer types [105]. Th17 cells take part in local inflammation producing IL-17 and IFN-*γ* and can therefore promote the inflammation-dependent tumor cell growth. Shifting the balance towards Th1 rather than Th17 differentiation resulted in a reduced population of Th17 cells, inhibited tumor inflammation, and reduced growth in a mouse pancreatic cancer model [106]. Moreover, Th17 cells and IL-17A cytokine were shown to promote angiogenesis in tumors [107], although other cytokines also produced by Th17 cells (IL-17F, IL-21, and IL-22) exhibited antiangiogenic properties [108–110]. It is therefore likely that Th17 subset can acquire different properties and cytokine production patterns depending on tumor microenvironment. Deeper understanding of Th17 regulatory mechanisms might allow harnessing these processes to fight the specific types of cancer.

## 5. Plasticity in Autoimmune Diseases

Loss of control of self-reactive T cells results in autoimmune diseases. Multiple sclerosis (MS) is an inflammatory disease of central nervous system caused by genetic variants combined with environmental factors, which is characterized by an abnormal activity of myelin-antigen reactive T cells [111]. Studies of EAE model of MS clearly demonstrated that IL-23/Th17 plays a key role in the disease pathogenesis [35, 112]. Moreover, GM-CSF has also been demonstrated to participate in the pathological process [113, 114] and elevated levels of IL-17 were found to be associated with the disease [115], pointing to an important role of Th17 cells in the MS pathogenesis, which has been also confirmed by a number of recent studies [116, 117]. Although the exact role of Th17 cells in the disease pathogenesis has not been elucidated so far, it has been proposed that these cells might be involved in the disruption of the blood-brain barrier [118]. Defects in Treg function have also been identified in MS as in other autoimmune diseases [49, 119].

The pathogenicity of Th17 cells in autoimmune diseases appears to be related to the Th17–Th1 plasticity, which is controlled by the cytokine environment [27]. Studies in the EAE model demonstrated that the pathogenic phenotype was characteristic specifically for Th17 cells generated in the presence of particular stimuli such as TGF-*β* and IL-23, underscoring the importance of T cell subset flexibility for the disease development [120, 121]. Importantly, CXCR3 ligands have recently been demonstrated to promote the polarization of naïve and effector T cells in EAE model. CXCL11 skewed the polarization of CD4+ T cells into Treg-like cells characterized by high production of IL-10, which resulted in suppression of EAE in IL-10-dependent manner. On the other hand, CXCL10 and CXCL9 promoted proinflammatory polarization of Th1 cells [86].

Treg plasticity was also shown to be implicated in the MS pathogenesis. Patients with relapsing remitting MS (RRMS) demonstrated significantly increased levels of IFN-*γ*-producing Th1-like Treg in peripheral blood [83]. Such increase could contribute to the loss of MS suppression observed in these patients [119]. A similar population of Th-1-like Tregs was reported in patients with type 1 diabetes as compared to healthy individuals [122]. Treg to Th17 conversion might also play a role in the pathogenesis of autoimmune disorders; Th17-like Treg cells have been observed in association with various autoimmune conditions [123, 124].

The correct functioning of Th subsets may depend on local microenvironment, which is most noticeable in skin diseases. Psoriasis is associated with cytokine imbalance in the skin with predominance of Th17 cytokines, IL-17, IL-21, IL-22, and TNF-*α* [68]. Treg to Th17 transition in psoriasis is driven by IL-23 signalling, and triple-positive CD4+/Foxp3+/IL-17A+ cells can be found in skin lesions. The regenerative role of IL-22 in the disease-affected skin turns upon its excess into a pathologic one, promoting skin thickening [125]. Accordingly, treatment aimed to correct the cytokine balance in the skin can play an important role in the therapy of psoriasis. Another skin disease, atopic eczema, is characterized by a Th2-dominated cytokine microenvironment, which antagonizes Th1 and Th17 immunity, resulting in local immune deficiency in the skin. Th2 cytokines also affect the epidermal barrier leading to skin dehydration [68]. In autoimmune hepatitis, a highly inflammatory microenvironment in the liver, enriched with IL-6, IL-17, IL-23, and IL-1*β*, tilted the balance towards Th17 rather than Treg differentiation, promoting the disease progression. Therefore, function of T cell cytokines is largely dependent on the cytokine content of the local microenvironment and disease setting, which has to be taken into account while developing therapy approaches aimed to adjust the Th subset imbalances [68].

## 6. Conclusion

T lymphocyte plasticity is an important mechanism that is likely evolved to enable the immune system to rapidly respond to the changing environment and adapt its functioning in the presence of infectious agents and parasites. Distinct subsets of T cells are regulated by a complex signalling network of cytokines and transcription factors, and disturbances in this network can cause serious pathologies, such as excessive inflammatory response in autoimmune diseases and enhanced immune tolerance in tumor microenvironment. Better understanding of these processes will allow development of novel therapeutic strategies based on reprogramming T cell populations towards one or another phenotype to reduce inflammation or enhance antitumor immunity.
